# Ceftriaxone- and ceftazidime-resistant *Klebsiella* species, *Escherichia coli*, and methicillin-resistant *Staphylococcus aureus* dominate caesarean surgical site infections at Mulago Hospital, Kampala, Uganda

**DOI:** 10.1177/2050312120970719

**Published:** 2020-11-10

**Authors:** Yvonne N Wekesa, Fatuma Namusoke, Musa Sekikubo, Dennis Wandera Mango, Freddie Bwanga

**Affiliations:** 1 Department of Obstetrics and Gynaecology, Makerere University College of Health Sciences, Kampala, Uganda; 2 MBN Clinical Laboratories, Kampala, Uganda; 3 Department of Medical Microbiology, Makerere University College of Health Sciences, Kampala, Uganda

**Keywords:** Caesarean section, surgical site infections, methicillin-resistant *Staphylococcus aureus* (MRSA), extended-spectrum beta-lactamase

## Abstract

**Objectives:**

The aim of this study was to determine the proportion and mechanism of resistance to ceftriaxone and ceftazidime among *Klebsiella* species and *Escherichia coli* and examine the burden of methicillin-resistant *Staphylococcus aureus* from caesarean section surgical site infections in Uganda.

**Methods:**

Wound swabs from 109 caesarean section surgical site infections were cultured for pathogenic bacteria following standard microbiological procedures. The Kirby–Bauer disc diffusion technique was used for antimicrobial susceptibility testing. Methicillin-resistant *S. aureus* diagnosis was based on polymerase chain reaction testing for the *mecA* gene. Data were analysed using SPSS-IBM Statistics v.20.

**Results:**

A total of 118 pathogens were recovered from 93 (85%) of 109 surgical site infections swabs. Of the 118 pathogens, gram-negative bacteria were 69 (58.5%), including 44 (37.3%) *Klebsiella* species, 11 (9.3%) *E. coli*, 6 (5.1%) *Citrobacter* species, and 8 (6.8%) other gram-negative bacteria. In total, 49 of the 118 pathogens were gram-positive bacteria, including 34 (28.8%) *S. aureus* and 15 (12.7%) *Enterococci* species. Resistance to ceftriaxone was detected in all 11 (100%) of the *E. coli* and in 43 (97.7%) of the 44 *Klebsiella* species and to ceftazidime in all 11 (100%) of the *E. coli* and 40 (91%) of the 44 *Klebsiella* species. Extended-spectrum beta-lactamase explained resistance to ceftazidime in 10 (91%) of the 11 *E. coli* and 19 (48%) of the 40 *Klebsiella* species. Carbapenemase production explained 15 (38%) of the 40 ceftazidime-resistant *Klebsiella* species. Methicillin-resistant *S. aureus* was detected in 91% of *S. aureus*.

**Conclusion:**

*Klebsiella* species, *E. coli*, and *S. aureus*–majority methicillin-resistant *S. aureus* dominated the pathogens in caesarean section surgical site infections. Almost all of the *E. coli* and *Klebsiella* species were resistant to ceftriaxone or ceftazidime. Extended-spectrum beta-lactamase was the underlying resistance mechanism among almost all of the ceftriaxone- or ceftazidime-resistant *E. coli.* However, this mechanism accounted for less than half of ceftriaxone- or ceftazidime-resistant *Klebsiella* species, where carbapenemases accounted for 40% of the resistance, a finding previously unreported in Uganda.

## Introduction

### Background

According to the World Health Organization (WHO), up to one-third of pregnancies end up as caesarean section (C/S) deliveries, making C/S one of the most common surgical procedures performed globally.^1^ Surgical site infections (SSIs), defined by not merely the presence of cultured microorganisms but also clinical signs of infection within 30 days after the C/S, are some of the commonest complications of C/S procedures worldwide.^2^ The worldwide incidence of SSI ranges from 0.5% to 26%.^3–6^ This wide variation in incidence of SSIs is reportedly due to varying infection control practices in the different global health facility settings. In sub-Saharan Africa, the incidence of SSI tends to be higher;^4^ for instance, in Kano, Nigeria, the incidence rate was estimated to be 9.1%.^4^ At Mulago National Referral Hospital in Kampala, Uganda, where around 500 C/S are performed monthly, there is hardly any reported data on the incidence of post-C/S SSIs. However, unpublished 2016 hospital records revealed that approximately 25–30 patients developed SSI monthly following C/S. If the infection is caused by drug-resistant pathogens, treatment with readily available antimicrobials may fail to eliminate the infection, potentially progressing to sepsis, a cause of maternal death in 10.7% and 30.9% of patients globally and in Uganda, respectively.^7,8^

While SSI is a common problem following C/S, limited data exist on the causative bacterial species and their susceptibility to antimicrobials at Mulago Hospital Uganda. This makes it difficult to accurately treat patients there, and in similar settings, where routine culture and antimicrobial susceptibility testing (AST) remain scarce. Studies conducted elsewhere have reported various findings regarding the bacterial aetiology and antimicrobial resistance profiles of the involved pathogens. A retrospective chart review of 191 patients in the United States in 2010 found methicillin-resistant *Staphylococcus aureus* (MRSA) as the most common organism isolated in post-C/S infections.^9^ Studies in Asia found the most common organisms in post-C/S SSIs to be *Escherichia coli, S. aureus*, and *Pseudomonas aeruginosa*, with high levels of MRSA and extended-spectrum beta-lactamase (ESBL) occurrence.^10,11^ A retrospective case–control study in Nigeria from 2001 to 2002 which examined post-C/S SSI found gram-negative bacteria (GNB) and *S. aureus* to be the most common pathogens, with 100% of GNB reported to be sensitive to cephalosporins.^4^ Recent studies in Tanzania and Rwanda found the most predominant pathogens in post-C/S SSIs to be *S. aureus* and *Klebsiella* species.^12,13^ Unlike in Tanzania, no MRSA was reported in the Rwanda study. However, these were prospective cohort studies conducted not only on post-C/S mothers who were symptomatic for SSIs but also on all post-C/S women. The only published study on bacterial aetiology of SSI at Mulago National Referral Hospital in Uganda was conducted almost 10 years ago, and it involved all SSIs, irrespective of the type of surgery. That study found *Escherichia coli* and *S. aureus* as the most common pathogens in 24% and 21% of SSIs, respectively;^14^ 75% of the *E. coli* were ESBLs, and 38% of *S. aureus* were MRSA. While that study examined SSI, it did not focus on post-C/S SSIs specifically, which forms the basis of this study.

In our study, we focused on only post-C/S SSIs. We set out to determine the dominant species of pathogenic bacteria, the proportion and mechanisms of resistance to ceftriaxone and ceftazidime (as representative agents of third-generation cephalosporins) among *E. coli* and *Klebsiella pneumoniae*, and the burden of MRSA isolated from post-C/S SSI at Mulago Hospital in Kampala, Uganda. The findings we report herein have potential for guiding empirical antibacterial treatment of post-C/S patients with clinical features of SSI, in hospitals with similar settings. Similar potential could be applied to practices where respective patients routinely receive combination prescriptions of ceftriaxone/metronidazole or piperacillin-tazobactam/metronidazole.

## Methodology

### Ethical consideration

Ethical permission to conduct the study was received from the School of Medicine Research and Ethics committee (REC REF: 2017-164) of Makerere University College of Health Sciences in Kampala, Uganda. Written informed consent was obtained from each study participant before recruitment into the study.

### Study design

This was a cross-sectional study conducted from November 2017 to April 2018.

### Study site and settings

The study was conducted at Mulago Hospital located in Kampala, Uganda. Mulago is a 1500-bed public hospital, and it operates as the national referral hospital for Uganda and the teaching hospital for Makerere University College of Health Sciences. The department of Obstetrics and Gynaecology is one of the departments at the hospital where about 15–25 C/Ss are carried out daily. Patients were recruited from the postnatal and gynaecology wards. All laboratory tests were conducted at MBN Clinical Laboratories, a centre of excellence in microbiology and molecular diagnostics in Kampala, Uganda

### Study population

Women who had undergone C/S and developed SSIs within 30 days, whether still admitted on the postnatal ward or as returnees on gynaecology wards, were recruited into the study.

### Sample size calculation

The sample size calculation was based on the prevalence of *S. aureus*–associated SSIs. The sample size calculation formula for a single proportion *N* = [*Z*^2^*p* (1 − *p*)]/*D*^2^ ^15^ was used, where *N* is the sample size, *Z* is the standard deviation value (1.96) corresponding to the 95% confidence interval, *p* is the estimated proportion of post-C/S mothers with SSIs caused by *S. aureus*, and *D* is the error margin, that is, 0.05. Based on a study in Tanzania among post-C/S mothers with SSI, the prevalence of *S. aureus* was 27.3%.^16^ By substituting these values in the formula, a sample size of 304 was obtained. However, since the average monthly number of post-C/S mothers with SSI at Mulago hospital is 25 and we conducted data collection over a period of 5 months, the accessible population would be 125 study participants. We, therefore, adjusted the sample size based on the sample size calculation formula for finite (known) populations,^17^ that is, *n*1 = *N*/(1 + {(*N* − 1)/Pop}] where, *n*1 is the required sample size, *N* is the estimated sample size from the first formula, that is, 304, and Pop is the finite population that we would have access to during the study period, that is, 125 mothers with clinical features of post-C/S SSI during the study period. By substituting values in the latter formula, we established a sample size of 88 study participants. By adding an additional 25% (i.e. 22 participants) of study participants to cater for possible non-response rate and other possible factors that could make the data incomplete, the estimated sample size was 110. We successfully recruited 109 mothers with post-C/S SSI.

### Inclusion criteria

Women with clinical features of post-C/S SSI, as defined under the Centers for Disease Control and Prevention (CDC) definition, were included in the study. The CDC defines post-C/S SSIs as presentation within 30 days of C/S with skin, subcutaneous tissue, fascia, muscle, or organ space having at least one of the following symptoms or signs: purulent discharge, pain/tenderness, local swelling, redness/heat, purulent discharge from drain, diagnosis of SSI by attending doctor, abscess revealed at clinical or radiological examination, or wound dehiscence, all with or without systemic symptoms of sepsis (*e.g*. fever, chills).^2^

### Exclusion criteria

Women with clinical features of post-C/S SSIs as defined above but who declined to participate in the study or who were too ill to consent were excluded.

### Sampling procedure

Consecutive sampling was employed, and all women meeting the inclusion criteria were recruited into the study. Four research assistants were trained on completion of the case report form (CRF)/data collection tool, collection of swab samples, swab storage, and transportation to the laboratory.

### Data collection

A CRF was used to extract data from each patient’s file. The information included demographic data, HIV status, length of preoperative hospital stay, indication for C/S, antimicrobial prophylaxis, antimicrobial empirical treatment, and other parameters as outlined in Table 1.

**Table 1.** Clinical characteristics of the study participants (*N* = 109).

Characteristic	Number	Percentage
HIV status		
Negative	104	95.4
Positive	5	4.6
Pre-operative stay (days)		
⩽2	60	55
>2	49	45
Presence of preoperative infection		
No	107	98.2
Yes	02	1.8
Type of caesarean		
Emergency	100	91.7
Elective	09	8.3
Antibiotic prophylaxis given		
Ceftriaxone	61	55.9
Both (ceftriaxone and metronidazole)	32	29.4
None	14	12.8
Metronidazole	02	1.8
Timing of antibiotic prophylaxis		
Pre-operation	10	9.2
Intra-operation	76	69.7
Post-operation	09	8.3
No information on patient chart	14	12.8
Presenting symptoms		
Discharge from the surgical site	100	91.7
Pain	53	48.6
Swelling	39	35.8
Wound dehiscence/gaping	32	29.4
Wound redness	22	20.2
Systemic symptoms of sepsis, for example, fever, chills	18	16.5
Indication for caesarean section		
Obstructed labour	46	42.2
Hypertensive disorder	12	11
Previous scar	19	17.4
Foetal distress	8	7.3
Obstructed labour and foetal distress	5	4.6
Other indications*	19	17.4

* Others included those indications with frequencies of two or less. These were ruptured uterus, big baby, cervical dystocia, cord prolapse, hydrocephalus, multiple pregnancy, ruptured uterus, severe oligohydramnios, persistent occiput posterior, malpresentation, and breech, all these either alone or in combinations.

### Specimen collection

Samples were taken from the patients during the period of surgical wound dressing, but before the wound was cleaned with antiseptic solution. Sterile cotton swabs soaked in sterile normal saline (0.9% NaCl) were used to wash out debris and to clean the surrounding skin before collecting the sample. A sterile swab was then used to collect any discharge from under the wound edges. Swabs were placed in gel Amies Transport Media,^18^ kept in a cool box without ice packs, and transported to the laboratory within 4–24 h.

### Laboratory procedures

#### Primary cultures

The swab specimens were processed at MBN Clinical Laboratories, located at 28 Nakasero Road, Kampala, Uganda. On receipt at the laboratory, the swabs were inoculated on Blood, MacConkey, and Chocolate agars and incubated at 35°C–37°C in an ambient incubator. If there was no growth on the agar plates within 72 h, the sample was declared negative for pathogenic bacteria. For the plates that showed growth of suspected bacterial pathogens, the bacteria were identified to genus and/or species levels.

#### Identification of bacterial pathogens

Identification of bacteria was performed based on colony characteristics, gram morphology and biochemical reactions as published in Cheesbrough et al.^19^ Colony characteristics included morphology, haemolysis on blood agar, changes in the physical appearance of colonies on differential media (e.g. a pink appearance of lactose-fermenting bacterial colonies on MacConkey agar) and gram stain morphology. GNB were identified based on colony characteristics such as mucoid colonies of *K. pneumoniae*, lactose fermentation, gram nature, motility and biochemical reactions such as on Triple Sugar Iron (TSI) agar, citrate, sulphur-indole motility (SIM) medium, urease, and oxidase tests. *S. aureus* was identified based on colony characteristics, gram-positive cocci with positive catalase and a slide/tube coagulase test. *Enterococcus* was identified based on gram-positive catalase-negative cocci and a positive bile-esculin test.

#### Antimicrobial Susceptibility Testing

**Figure 1.**
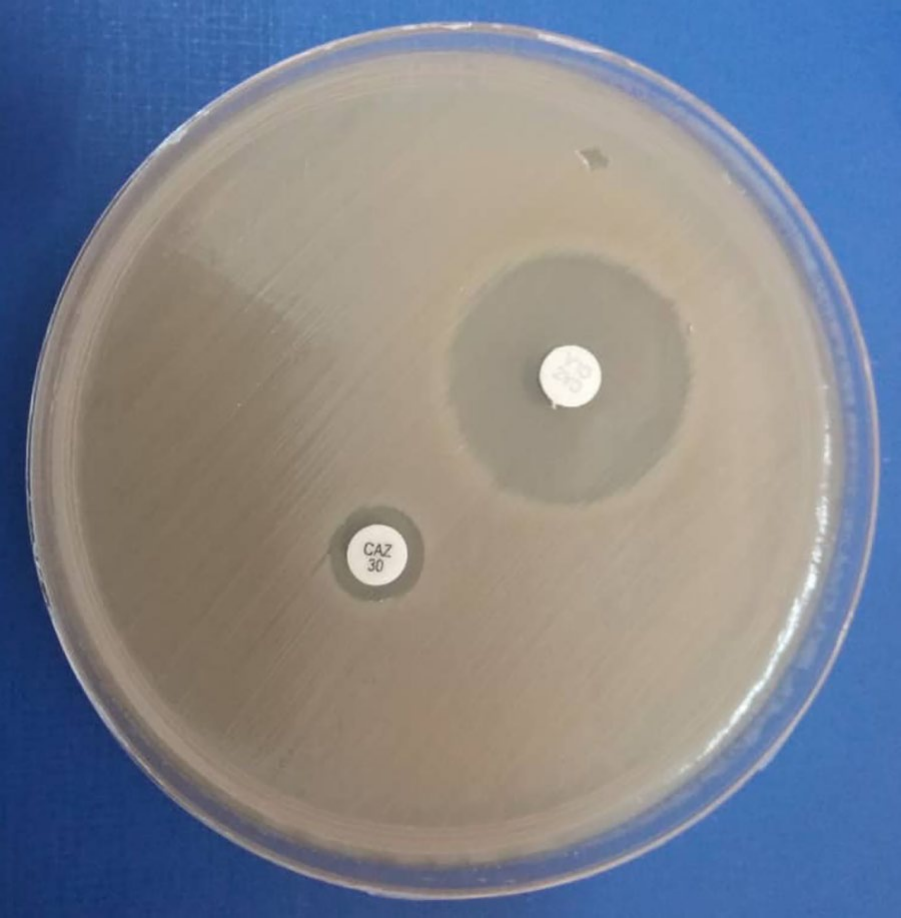
ESBL confirmation in *E. coli.* *Left*: Ceftazidime (CAZ) 30 μg/mL disc where the zone diameter was 9 mm, indicating resistance to ceftazidime. *Right*: Ceftazidime/clavulanate (30/10 μg) disc with an enhanced growth inhibition zone diameter of 24 mm, that is, at least 5 mm diameter increase, confirming the *E. coli* as an ESBL strain.

AST was based on the Kirby–Bauer disc diffusion methods.^20^ The methods, the choice of tested antimicrobials per organism, and disc concentrations were selected according to the Clinical and Laboratory Standards Institute (CLSI) guidelines to the extent possible.^21^ Briefly, the inoculum was prepared and standardised in sterile normal saline against a 0.5 McFarland solution. A sterile swab was dipped into the prepared inoculum suspension, squeezed against the side of the tube to get rid of excess fluid, and then spread evenly over the surface of the Mueller–Hinton agar plate. Antibacterial discs were then placed onto the inoculated plates and incubated at 35°C–37°C for 16–18 h.^22^ After this period, growth inhibition zone diameters were measured to the nearest millimetre, using a ruler. GNB pathogens were tested against ampicillin (10 μg), amoxicillin/clavulanate (20/10 μg), cefuroxime (30 μg), ceftriaxone (30 μg), ceftazidime (30 μg), gentamicin (10 μg), tetracycline (30 μg), ciprofloxacin (5 μg), trimethoprim/sulfamethoxazole (1.25/23.75 μg), chloramphenicol (30 μg), imipenem/meropenem (10 μg), and amikacin (30 μg). ESBL production as the mechanism of underlying resistance to ceftriaxone or ceftazidime in *E. coli* and *Klebsiella* species was screened based on the disc diffusion test when zone diameters of ceftazidime (30 μg) alone were compared with ceftazidime/clavulanate (30/10 μg) zone diameters on Mueller–Hinton agar.^21^ An enhanced inhibition zone diameter of at least 5 mm around the ceftazidime/clavulanate (30/10 μg) disc relative to ceftazidime alone indicated positive ESBL production in that particular bacterial pathogen as shown in Figure 1.

If no zone diameter increase occurred, we considered it to be a non-ESBL mechanism of resistance to ceftazidime, which could be ampC or carbapenamase production, as neither of these two is inhibited by clavulanic acid. If a non-ESBL ceftazidime-resistant organism was found to be susceptible to carbapenems, ampC was considered as the resistance mechanism to the ceftazidime. However, if the organism was resistant to carbapenems, then carbapenamase production was considered as the mechanism of resistance to ceftazidime.

**Figure 2.**
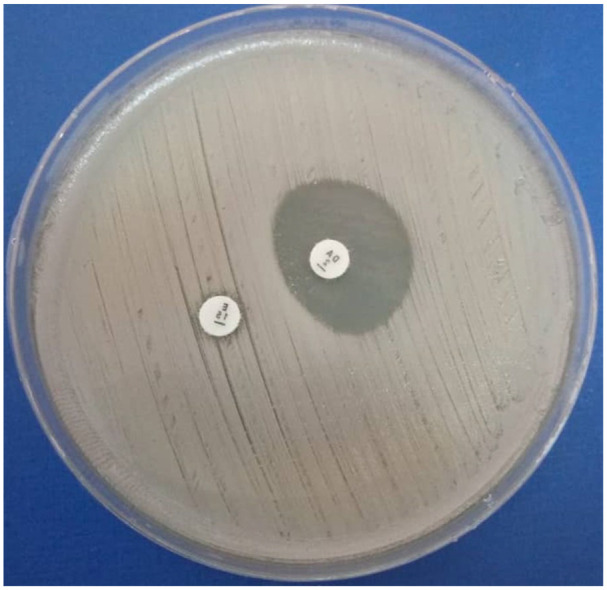
Inducible resistance to clindamycin (DA) in *S. aureus* caused by erythromycin (E). Note the flattening around the DA disc on the side nearest to the E disc, resulting in formation of a D-shaped zone around the DA disc.

For gram-positive organisms, susceptibility was tested against penicillin (10 units), vancomycin (30 μg), linezolid (30 μg), gentamicin (10 μg), erythromycin (15 μg), tetracycline (30 μg), ciprofloxacin (5 μg), clindamycin (2 μg), trimethoprim/sulfamethoxazole (1.25/23.75 μg), and chloramphenicol (30 μg). Induced clindamycin resistance among *S. aureus* was detected based on the double disc diffusion method^23^ on Mueller–Hinton agar as follows: an erythromycin disc (15 μg) and a clindamycin disc (2 μg) were spaced 15–20 mm apart and incubated at 35 °C ± 2°C for 16–18 h. Flattening of the zone of inhibition adjacent to erythromycin (D-zone), that is, a positive D-test, was interpreted as inducible clindamycin resistance as shown in Figure 2.

MRSA was identified based on polymerase chain reaction (PCR) testing for the *mecA* gene as described below.

### MRSA confirmation with *mecA* PCR

#### DNA extraction

The boiling method was used to extract chromosomal DNA from *S. aureus*.^24^ Briefly, 3–5 mature colonies of *S. aureus* were harvested and emulsified in 300 mL of phosphate-buffered saline (PBS) in a cryo vial, heated at 100°C for 30 min, and then centrifuged at 4000 r/min in a microcentrifuge. The supernatant was aliquoted into a new Eppendorf tube ready for PCR and kept at −20°C.

#### Primers

Previously published forward primer P4: 5′-TCCAGATTACAACTTCACCAGG-3′ and reverse primer P7: 5′-CCACTTCATATCTTGTAACG-3′, which amplify a 162-bp segment of the *mecA* gene, were used in this study.^25^ These primers were procured from Integrated DNA Technologies.^26^ The primer concentration for each of primers P4 and P7 was optimised at 100 ng/µL, and 0.5 µL (*i.e.* 50 ng) of each of these primers was added to each PCR reaction mix, consisting of RNAse-free water (8 µL), master mix (1 µL) containing dNTPs and MgCl_2_, and Taq polymerase (0.1 µL). Extracted DNA was then added at 1 µL, and the total reaction volume was 11.1 µL.

#### Amplification parameters for the *mecA* gene

These parameters included initial denaturing at 94°C for 4 min, followed by 30 cycles of denaturing at 94°C for 30 s, primer annealing at 53°C for 30 s, extension at 72°C for 60 s, and a final elongation step of 4 min at 72°C in the Gene Amp PCR System 9700 Thermocycler (Applied Biosystems Inc., Foster City, California, USA).

#### Electrophoresis

**Figure 3.**
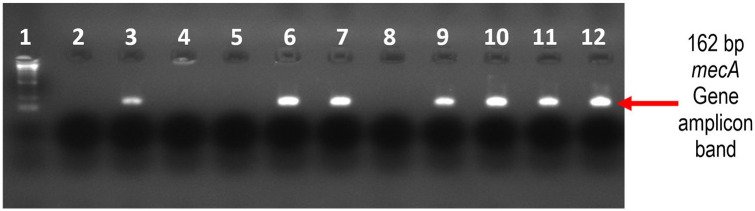
Gel electrophoresis image of the *mecA* gene. Lane 1: 100-bp DNA Ladder; Lane 2: blank with PCR reagents only; Lane 3: *mecA*-positive control (ATCC 43300); Lane 4: *mecA*-negative control (ATCC 29213); Lanes 5 and 8: *S. aureus* isolates negative for the *mecA* gene; Lanes 6, 7 and 9–12: *S. aureus* isolates positive for the *mecA* gene.

This was conducted on 2% agarose stained with 10 µL of ethidium bromide 5 μg/mL. A 100-bp DNA ladder was used as a molecular weight marker. A DC voltage of 120 was used during electrophoresis for 45–60 min. DNA bands were photographed using a digital UV camera, and results were interpreted as *mecA-positive* where a 162-bp band was observed, as shown in Figure 3.

#### Quality assurance

All culture media were prepared according to the directions of the manufacturers.^22^ Three plates of each batch were incubated at 37°C for 48 h to check for sterility. The ability to support the growth of the common organisms causing SSI was determined by inoculating the media with typical stock cultures of *S. aureus, E. coli* and *K. pneumoniae*, pre-stored at the laboratory. Negative and positive controls were included to validate the biochemical reagents/test kits. Standard quality control (QC) strains were used to monitor the accuracy and precision of susceptibility testing procedures, antibiotic discs, as well as performance of the person carrying out the test and reading the results. *S. aureus* ATCC 25923 was used as QC strain for biochemical identification of *S. aureus. E. coli* ATCC 25922 was used as QC strain for biochemical identification and disc diffusion AST of *E. coli. P. aeruginosa* ATCC 27853 was used as QC strain for biochemical identification and disc diffusion AST of *P. aeruginosa.* In-house strains of ESBL-positive *E. coli* and *K. pneumoniae* were used as QC strains during identification and disc diffusion AST of *E. coli* and *K. pneumoniae.* For the *mecA* PCR tests, each PCR and electrophoresis batch was controlled with plain PCR reagents (no DNA), known *mecA*-positive *S. aureus* strain (ATCC 43300), and known *mecA*-negative *S. aureus* strain (ATCC 29213).

#### Statistical data analysis

Before data entry, the CRFs and laboratory results were reviewed for completeness, consistency and accuracy. Queries were resolved and then the data were entered, checked again, and analysed using SPSS-IBM Statistics v.20.^27^ Categorical variables were presented using proportions or percentages. Continuous variables were summarised using interquartile ranges, means, medians, and standard deviations. Calculations for statistical significance of the proportions were performed based on 95% confidence interval estimation for binomial proportions, and the asymptotic (Wald) normal approximation method was used.^28^

## Results

### Clinico-demographic characteristics of studied participants

The study enrolled 109 patients with clinical SSIs following C/S. The median age was 25 years (interquartile range, 10 years; minimum age, 16 years; maximum age, 41 years). In total, 95% of the patients were HIV negative and 94% stayed in the hospital for up to 3 days prior to the operation. There was no clinical evidence of preoperative infection in 98% of the studied participants. Ninety-two percent (100/109) of the cases had emergency C/Ss, and 87% had received antibiotics (ceftriaxone and/or metronidazole) perioperatively, as detailed in Table 1. The most commonly presented complaint among the study participants was discharge from the wound, with 92% of participants reporting its occurrence. Other forms of presentation included pain, swelling, wound dehiscence, redness, and systemic symptoms in 49%, 36%, 29%, 20%, and 17% of patients, respectively. Most of the patients displayed a combination of these, as shown in Table 1. The most common indications for C/S requirement included obstructed labour, previous scar, hypertensive disorder, and foetal distress in 42% (46/109), 17% (19/109), 10.2% (12/109), and 7% (8/109) of patients, respectively. Some patients had multiple indications requiring C/S. Details of indications for C/S are shown in Table 1.

### Bacterial species isolated from post-caesarean SSIs

Of the 109 swabs, 93 (85%) were culture-positive with one (68 samples) or more (25 samples) pathogenic bacteria, giving a total of 118 bacterial pathogens. Seven samples demonstrated the growth of common skin contaminants (*Bacillus* species and coagulase-negative staphylococci), while nine samples did show any growth. Of these 118 bacterial pathogens, 69 (59%) were GNB and 49 (41%) gram-positive bacteria (GPB). Among the 69 GNB, the most predominant species was the *Klebsiella* species, present in 44 (63.8%) of the samples, followed by *E. coli* in 11 (15.9%). Among the 49 GPB pathogens, *S. aureus* contributed 34 (69.4%), while *Enterococci* species accounted for 15/118 (30.6%) pathogens. Details of the isolated bacterial species are shown in Table 2.

**Table 2.** Number and percentage of bacterial species isolated from post-caesarean surgical site infections.

Organism	Frequency, out of total pathogens (*n* = 118)		Frequency, out of gram-negatives (*n* = 69)	
GNB (*n* = 69)	*n* (%)	95% confidence interval	*n* (%)	95% confidence interval
*Klebsiella* species (*K. pneumoniae* (43) and *K. oxytoca* (1))	44 (37.4)	37–46	44 (63.8)	52–75
*Escherichia coli*	11 (9.3)	4–15	11 (15.9)	7–25
*Citrobacter* species	6 (5.1)	1–9	6 (8.7)	2–15
*Acinetobacter* species	5 (4.2)	1–8	5 (7.2)	1–13
*Enterobacter* species	2 (1.7)	−1–4	2 (2.9)	−1–7
*Pseudomonas aeruginosa*	1 (0.8)	−1–3	1 (1.4)	−1–4
Subtotal	69 (59.0)	50–67	69 (100.0)	100–100
GPB (*n* = 49)	Frequency, out of total pathogens (*n* = 118)		Frequency, out of gram-positives, (*n* = 49)	
	*n* (%)	95% confidence interval	*n* (%)	95% confidence interval
*Staphylococcus aureus*	34 (28.8)	21–37	34 (69.4)	56–82
*Enterococcus* species	15 (12.7)	7–19	15 (30.6)	18–44
Subtotal	49 (41.0)	33–50	49 (100.0)	100–100
Overall total	118 (100.0)	100–100	49 (100.0)	100–100

GNB: gram-negative bacteria.

### Antibacterial resistance

#### Resistance to ceftriaxone and ceftazidime among GNB

Resistance to ceftriaxone was identified in all 11 of the *E. coli* isolates (100%) and in 43 (98%) of the 44 of *Klebsiella* species. Resistance to ceftazidime was found in all 11 (100%) of the *E. coli* isolates and in 40 (90.9%) of the 44 *Klebsiella* species. One (2.2%) of the 44 *Klebsiella* species showed intermediate susceptibility, while only 3 (6.8%) were susceptible to ceftazidime. Additional antimicrobial resistance data on the other drugs and other GNB are shown in Table 3.

**Table 3.** Percentage antibacterial resistance among gram-negative bacterial pathogens (*N* = 69).

Antibacterial agents	*Klebsiella* species (*n* = 44), %	*Escherichia coli* (*n* = 11), %	*Citrobacter* species (*n* = 06), %	*Acinetobacter* species (*n* = 05), %	*Enterobacter* species (*n* = 02), %	*Pseudomonas aeruginosa* (*n* = 01), %
Ampicillin	100.0	100.0	100.0	NA	100.0	NA
Amoxicillin/clavulanic acid	95.5	100.0	83.3	NA	50.0	NA
Ceftriaxone	97.7	100.0	83.3	NA	50.0	NA
Ceftazidime	90.9	100.0	66.7	60.0	50.0	00.0
ESBL	43.2	90.9	NA	NA	NA	NA
Carbapenems	34.1	00.0	00.0	40.0	00.0	00.0
Chloramphenicol	68.2	27.3	66.7	NA	50.0	NA
Ciprofloxacin	47.7	81.8	50.0	60.0	00.0	00.0
Trimethoprim/sulfamethoxazole	93.2	100.0	100.0	100.0	50.0	NA
Gentamicin	77.3	63.6	50	60.0	50.0	00.0
Tetracycline	NA	NA	NA	40.0	NA	NA
Amikacin	00.0	00.0	00.0	00.0	00.0	00.0

ESBL: extended-spectrum beta-lactamase; NA: drug not recommended for testing in that bacterial species or not tested due to lack of stock.

#### Mechanism of resistance to ceftriaxone and ceftazidime among *E*

*coli* and *Klebsiella* species.

Among the 11 ceftazidime-resistant *E. coli*, ESBL production was the underlying mechanism in 10 (91%), and all 10 of these were susceptible to carbapenems. Among the 40 ceftazidime-resistant *Klebsiella* species, ESBL production was the underlying mechanism in 19 (47.5%), and all were susceptible to carbapenems. No ESBL enzyme production could be detected in the other 21 ceftazidime-resistant isolates. In these, we found 6 (15%) to be susceptible to carbapenems, suggesting ampC beta-lactamase production as the underlying mechanism of resistance to ceftazidime, and the remaining 15 (37.5%) isolates to be resistant to carbapenems, suggesting carbapenemase enzyme production as the underlying mechanism of resistance to ceftazidime and to the carbapenems themselves. Detailed resistance data are shown in Table 3.

### Resistance among GPB

Among the 34 *S. aureus* pathogens, 33 (97%) were resistant to penicillin and 31 (91.2%) were identified as MRSA. Inducible resistance to clindamycin by erythromycin (D-test positive) was found in 14 (41%) of the 34 *S. aureus* pathogens. However, 13 (93%) of the 14 *Enterococci* pathogens were susceptible to penicillin. All of the GPB (34 *S. aureus* and 15 *Enterococci*) were susceptible to vancomycin, and all the 15 *Enterococci* were susceptible to linezolid. Detailed data on resistance among GPB are shown in Table 4.

**Table 4.** Percentage of antibacterial resistance among gram-positive bacterial pathogens (*N* = 49).

Antibacterial agent	*Staphylococcus aureus* (*n* = 34), %	*Enterococcus* species (*n* = 15), %
Penicillin	97.1	6.7
MRSA	91.2	NA
Trimethoprim/sulfamethoxazole	97.1	NA
Ciprofloxacin	50.0	20.0
Gentamicin	52.9	NA
Erythromycin	70.6	80.0
D-test positive	58.8	NA
Tetracycline	44.1	86.7
Chloramphenicol	20.6	40
Vancomycin	00.0	00.0
Linezolid	NA	00.0

MRSA: methicillin-resistant *Staphylococcus aureus*, NA: drug not recommended for testing in that bacterial species or not tested due to lack of stock.

### Resistance of MRSA to other antimicrobials

Over 50% of the MRSA isolates were also resistant to any of sulfamethoxazole/trimethoprim (SXT), erythromycin, ciprofloxacin, or gentamicin. All MRSA isolates were susceptible to vancomycin. Details of resistance to other antimicrobials among MRSA isolates are shown in Table 5.

**Table 5.** Number and percentage susceptibility profile of MRSA isolates to other antimicrobials (*n* *=* 31)..

Antimicrobial agent	Resistant, *n* (%)	Intermediate, *n* (%)	Susceptible, *n* (%)
Sulfamethoxazole/trimethoprim	30 (96.8)	0 (0.0)	1 (3.2)
Erythromycin	23 (74.2)	2 (6.5)	6 (19.4)
Ciprofloxacin	17 (54.8)	1 (3.2)	13 (41.9)
Gentamicin	17 (54.8)	2 (6.5)	12 (38.7)
Tetracycline	14 (45.2)	0 (0.0)	17 (54.8)
Inducible clindamycin resistance (D-test Positive)	13 (41.9)	0 (0.0)	18 (58.1)
Chloramphenicol	6 (19.4)	1 (3.2)	24 (77.4)
Vancomycin	0 (0.0)	0 (0.0)	31 (100)

## Discussion

SSIs remain common complications of C/S, affecting approximately 25–30 patients monthly at the Mulago National Referral Hospital in Kampala, Uganda. Proper clinical management of these infections should rely on the use of antimicrobials, prescribed on the basis of routine culture and susceptibility results, or on statistical surveillance data relating to the dominant pathogens and their susceptibility profiles. In the absence of both, as is the case at Mulago and other hospitals in the resource-limited settings of sub-Saharan Africa, antimicrobial prescriptions for patients with SSI are usually empirical. The data herein provide up-to-date information on the dominant pathogens in post-C/S SSIs and their susceptibility profiles, including resistance mechanisms to key selected groups of antimicrobials, as a basis for guiding empirical antimicrobial prescriptions in the relevant clinical settings.

Our findings show that GNB were the most common pathogenic bacteria in post-C/S SSIs, causing 6 out of every 10 post-C/S SSIs with *K. pneumoniae* and *E. coli* being most prevalent. Our findings are similar to those reported in a study by Seni et al.^14^ in 2012, who identified gram-negative enteric bacterial pathogens in about 68% of SSI cases, although their study involved swabs from post-surgical infections following many procedures, not only C/S. Another study of post-C/S SSIs which took place in Ethiopia also identified GNB in 60% of samples.^29^

Among the GPB, there were only two pathogenic species, with *S. aureus* dominating. Other GPB, such as group A streptococci, were not detected in the examined SSIs, most likely because they are typically sensitive to beta-lactam antibiotics, and most of the patients had received ceftriaxone as prophylactic treatment prior to, or just after, surgery. Seni et al.^14^ in 2012 found *S. aureus* in 20.4% of all SSIs, and another study carried out in Ethiopia identified *S. aureus* as the causative pathogen in 23.4% of SSIs.^29^ Another study in Rwanda reported *S. aureus* as the dominant pathogen in 62.5% of organisms, although this study looked at only 16 samples.^12^ Several other studies also found *S. aureus* to be a dominant pathogen in post-C/S SSIs.^3,4,12^ Other studies also reported multiple pathogens recovered through culture of swabs from post-C/S SSIs. In our study, 25 (27%) of the 93 swabs resulted in the growth of more than one pathogen, a finding only slightly lower than the 37% multiple-pathogen recovery from swabs reported in a 2012 Mulago hospital study, among SSIs from all surgical wards at the hospital.^14^ From the above discussion, it appears that proper management of post-C/S SSIs with antimicrobial agents requires prior culture and susceptibility testing and use of more than one antimicrobial drug of reasonable efficacy, with low resistance rates, targeting gram-negative *Enterobacteriaceae, S. aureus* and *Enterococcus*.

Considering antimicrobial resistance in relation to the limited resources allocated to patients attending Mulago and similar hospitals in Africa, it is worrying that all GNB were found to be resistant to the otherwise readily available drugs, such as ampicillin and amoxicillin/clavulanic acid, and to the commonly used third-generation cephalosporins, ceftriaxone, or ceftazidime. High percentages of resistance to ampicillin (90%–100%) among GNB pathogens, as detected in our study, have also been reported in several studies carried out in Africa, including Ethiopia, Tanzania and Uganda.^14,29,30^ In addition, the same studies reported that over 90% of these gram-negatives were also resistant to amoxicillin/clavulanic acid (augmentin), which is another readily available drug on the essential drug lists of hospitals in sub-Saharan Africa. Furthermore, the resistance to third-generation cephalosporins, particularly ceftriaxone or ceftazidime, in all *E. coli* and in over 90% of *Klebsiella* species is one of the greatest clinical challenges since these drugs are the most extensively prescribed agents for empirical treatment of SSIs in these resource-limited settings. A study in 2011–2012 by Seni et al.^14^ found resistance to these third-generation cephalosporins in 78% of *E. coli* and 87% of *Klebsiella* species. Thus, it appears that this resistance prevalence is increasing in Uganda.

In clinical settings, the mechanism of resistance to ceftriaxone or ceftazidime (third-generation cephalosporins) underpins the subsequent choice of drug for use in the treatment of patients with SSIs. Of the 11 *E. coli* strains that were resistant to ceftazidime, 10 (91%) were ESBL producers and thus potentially treatable with a third-generation cephalosporin combined with a beta-lactamase inhibitor, such as clavulanate, since ESBL enzymes are conventionally inhibited by clavulanate, or with carbapenems, since these agents are not hydrolysed by ESBL enzymes. However, this would not be the case with *Klebsiella* species, where only 19 of the 40 ceftazidime-resistant isolates were ESBL producers, and 15 (37.5%) of the other 21 showed additional resistance to carbapenems. The actual mechanism of resistance to ceftazidime among these 15 isolates was, therefore, most likely due to the production of carbapenemases since these isolates showed phenotypic resistance to carbapenems. For the remaining six isolates, the mechanism was most likely due to ampC beta-lactamase production or due to some other mechanisms that were not studied here.^31^ It appears that the burden of resistance to carbapenems is increasing among the *Klebsiella* species in Uganda. Our claim is supported by data reported in a study by Seni et al.^14^ in 2012, which did not find any *Klebsiella* species isolated from SSIs to be resistant to carbapenem agents. However, in our study, conducted 6 years later, we found 15 isolates to be carbapenem-resistant. Carbapenem antimicrobial agents have been used as a last-resort, salvage treatment option for infections caused by multidrug-resistant gram-negative bacteria (MDR-GNB). Infections caused by carbapenem-resistant GNB are extremely difficult to treat, and the overall 30-day mortality in the case of systemic infections has been reported up to 50%.^32,33^ Whereas SSIs are largely local wound infections, some do progress to systemic infections, and if the causative agent is a carbapenem-resistant organism, the risk of mortality can be high.

Among the GPB, the most worrying form of resistance is MRSA. MRSA occurs when *S. aureus* acquires a novel gene known as the *mecA* gene, which encodes for an altered penicillin-binding protein (PBP2A) with very low affinity for the beta-lactam ring of beta-lactam antibacterial agents.^34^ This confers resistance to almost all beta-lactam agents, including third-generation cephalosporins and carbapenems, the exception being ceftaroline and other newer, expensive, and difficult to access cephalosporins.^35^ Therefore, our finding of MRSA resistance in 9 of every 10 *S. aureus* pathogens from post-C/S SSIs is extremely worrying. Our study revealed a very high MRSA prevalence compared to findings in the previous studies in Uganda, which found MRSA resistance in only 30%–40% of *S. aureus*,^3,14,29,30,36^ again suggesting that the burden of MRSA is increasing in Uganda.

The reasons for the worsening state of resistance remain poorly studied in the local settings. However, the use of the hitherto broad-spectrum drugs, such as ceftriaxone, for prophylaxis, and other instances of inappropriate use of such agents, has been suspected as one of the underlying causes of resistance. A meta-analysis study that involved 51 randomised controlled trials reported that ampicillin and first-generation cephalosporins have similar efficacy in prophylactic treatment of SSI.^37^ According to the Clinical Practice Guidelines for Antimicrobial Prophylaxis in Surgery, cefazolin, a first-generation cephalosporin, is the drug recommended for primary prophylaxis in C/S surgeries since it is more specific with a narrower antibacterial spectra, in addition to it being an inexpensive product.^38^ Furthermore, an observational prospective cohort study in 2018 at a tertiary hospital in Thailand reported no difference between ampicillin and ceftriaxone in the prevention of SSIs after C/S and recommended the use of ampicillin for prophylactic treatment in C/S patients.^39^ In Mulago and many hospitals in Uganda and sub-Saharan Africa, almost all patients undergoing C/S surgical procedures are given intravenous ceftriaxone, with or without metronidazole, either pre-, intra- or post-operatively, a practice which is probably based on drug availability in hospitals. In this study, contrary to the recommendations above, all except 14 patients (for whom we could not obtain information) received antimicrobial prophylaxis with ceftriaxone and/or metronidazole, as shown in Table 1. Ceftriaxone is reported not to be appropriate for surgical prophylaxis because of its pharmacokinetic profiles and the fact that being a broad-spectrum drug, its overuse can exert a selective pressure that quickly results in the emergence of multidrug-resistant organisms.^40^ It is, therefore, possible that the high proportion of resistance to ceftriaxone or ceftazidime, as reported in our study, could be due to selective pressure occasioned by the uncontrolled and often inappropriate use of these agents in Uganda’s health facilities for over 10 years.

In addition, we found that ceftriaxone and metronidazole were routinely used to treat post-C/S SSIs without prior culture and susceptibility testing data, and this has been the standard practice in the study clinical settings. Due to the high resistance rates reported in our study, it is possible that the use of these drugs to treat post-C/S patients with SSIs caused mainly by *E. coli* or *Klebsiella* species is not a recommended practice. Since ESBL resistance mechanisms were the most prevalent causes based on our data, the use of these third-generation cephalosporins, in combination with a beta-lactamase inhibitor such as sulbactam, could be considered.

Furthermore, our finding of MRSA in over 90% of cases where the use of ceftriaxone and metronidazole for C/S SSI treatment was employed clearly demonstrates that there is little logic to this practice since all MRSA strains are resistant to all beta-lactams, including ceftriaxone. This may imply that the treated SSI patients who eventually recovered did so due to wound care, patient immunity and possibly metronidazole, which has been shown in previous in vitro studies to kill MRSA,^41^ although susceptibility of MRSA to this drug was not part of our study. Infections due to MRSA can only be treated with other chemical classes of antimicrobials (not beta-lactams). If infections are local, such as the case in this study, oral treatment would be preferred. However, as shown in Table 5, most MRSA isolates were resistant to the commonly available oral antimicrobials. Patients with systemic infections arising from SSIs would require vancomycin, which demonstrated a 100% susceptibility rate, as also reported in other studies.^3,14,30^

Considering all of the above, there is an urgent requirement for a review of the guidelines for C/S antimicrobial prophylaxis and first-line antibacterial treatment of post-C/S SSI in settings such as Mulago Hospital. Patients with SSIs who routinely receive ceftriaxone and metronidazole probably improve not because of antibacterial drug treatment, but due to wound hygiene and their own immunity. From our study, ciprofloxacin (to which approximately half of *Klebsiella* species or *S. aureus* were susceptible), chloramphenicol (to which approximately three-quarters of *E. coli* and *S. aureus* were susceptible), and penicillin (to which 93% of *Enterococcus* were susceptible) would appear to be the drug combination of choice for oral treatment of SSIs, as no other effective options appear to be available. However, ciprofloxacin, a quinolone, is not recommended in breast-feeding mothers due to the possible adverse effects in skeletal tissues of the newborns/children less than 8 years of age. Chloramphenicol, if given orally, is also associated with severe nausea and vomiting and may also cause bone marrow suppression. This leaves clinicians with a very narrow choice for oral treatment of SSIs, a fact that calls for enhanced combined efforts in the control and prevention of the spread of antimicrobial resistance.

For systemic infections arising from SSIs, a third-generation cephalosporin combined with (1) a beta-lactamase inhibitor, carbapenems, or amikacin to cover gram-negatives and (2) vancomycin for GPB appears to be the best choice of antibacterial treatment. However, it should be noted that amikacin can cause both autotoxicity and renal toxicity.

## Limitations

The sample size of 109 participants is probably not adequate to make a conclusive observation of the problem of drug resistance in post-C/S SSIs. Nevertheless, the data reported herein demonstrate significant implications relating to drug resistance in this group of patients. Some drugs such as piperacillin-tazobactam could not be tested on all GNB pathogens, neither could we do cefoxitin on *S. aureus* in this study due to procurement predicaments, but we did the *mecA* PCR, which is a recognised method for MRSA diagnosis.

## Conclusion

*Klebsiella* species, *E. coli* and *S. aureus–*majority MRSA, dominated the pathogens involved in causation of C/S SSIs at the Mulago National Referral Hospital in Kampala, Uganda. Almost all of the *E. coli* and *Klebsiella* species were resistant to ceftriaxone or ceftazidime. ESBL was the underlying resistance mechanism among almost all the ceftriaxone- or ceftazidime-resistant *E. coli*, but this mechanism related to less than half of ceftriaxone- or ceftazidime-resistant *Klebsiella* species, where carbapenemase production caused closer to 40% of the resistance, a worrying finding previously unreported in Uganda. The clinical implication of our findings is that majority of post-C/S SSIs in Mulago National Referral Hospital may not be treatable using ceftriaxone or ceftazidime alone but potentially treatable with third-generation cephalosporins combined with a beta-lactamase inhibitor to block ESBLs but more than half of the ceftazidime-resistant *Klebsiella* species could not be managed this way since many out-rightly demonstrated carbapenemase production as mechanism of resistance to the ceftriaxone, ceftazidime and carbapenems themselves. Among the *S. aureus* pathogens, 9 of every 10 strains were MRSA and thus not treatable with almost all beta-lactam agents, yet many were also resistant to other antimicrobial classes. We strongly recommend routine culture and AST on post-C/S SSIs to optimise antimicrobial regimens for each patient.

## Supplemental Material

CASE_REPORT_FORM_WEKESA_et_al_Dr._Bwanga_17July2020 – Supplemental material for Ceftriaxone- and ceftazidime-resistant Klebsiella species, Escherichia coli, and methicillin-resistant Staphylococcus aureus dominate caesarean surgical site infections at Mulago Hospital, Kampala, Uganda Click here for additional data file. Supplemental material, CASE_REPORT_FORM_WEKESA_et_al_Dr._Bwanga_17July2020 for Ceftriaxone- and ceftazidime-resistant Klebsiella species, Escherichia coli, and methicillin-resistant Staphylococcus aureus dominate caesarean surgical site infections at Mulago Hospital, Kampala, Uganda by Yvonne N Wekesa, Fatuma Namusoke, Musa Sekikubo, Dennis Wandera Mango and Freddie Bwanga in SAGE Open Medicine
