# Supplementary material for: Ceftriaxone- and ceftazidime-resistant Klebsiella species, Escherichia coli, and methicillin-resistant Staphylococcus aureus dominate caesarean surgical site infections at Mulago Hospital, Kampala, Uganda
Source: SAGE Open Med. 2020 Nov 10;8:2050312120970719. doi: 10.1177/2050312120970719 (PMC8826261; doi:10.1177/2050312120970719)
Supplement: CASE_REPORT_FORM_WEKESA_et_al_Dr._Bwanga_17July2020 – Supplemental material for Ceftriaxone- and ceftazidime-resistant Klebsiella species, Escherichia coli, and methicillin-resistant Staphylococcus aureus dominate caesarean surgical site infections at Mulago Hospital, Kampala, Uganda [file CASE_REPORT_FORM_WEKESA_et_al_Dr._Bwanga_17July2020.docx]

**CASE REPORT FORM**

**STUDY TITLE:** BACTERIAL SPECIES AND ANTIBACTERIAL RESISTANCE AMONG POST CAESEREAN SECTION SURGICAL SITE INFECTIONS AT MULAGO HOSPITAL

Most of the information will be extracted from patients clinical case notes (patients’ files)

**Indicates information’s gathered directly from the patients*

Serial Study number………………………………………..

Date of interview ……………………….Hospital Registration number …………………………

Tick Sample taken;

SWAB……………………………

SYRINGE……………………….

**1. Ward or clinic in which patient admitted or attending**

**2. Age…………………………… 3. Address………………………..**

**4. Date of Admission……………………..**

**5. Date of surgery……………6. Date of discharge……………**

**7. History of previous use of antibiotics within one month**

(a) Yes

(b) No

**8. If yes, write names of the antibiotics used**

**9. For how long have been on such antibiotics**

(a) <=7 days

(b) 8-15 days

**10. Preoperative hospital stay:**

(a) <= 3 days (b) 4-7 days (c) More than 7 days

**11. Presenting complains –TICK ALL APPLICABLE**

(a) Pain /tenderness

(b) Swelling at the operation site

(b) Gaping at the operation Site

(c) Discharge from surgical site * /or from drain

(d) Redness or heat

(e) Systemic symptoms of sepsis-fever, chills

(f) Radiological

**12. Past Medical History**

(a) DM

(b) Prolonged Steroid usage

(c) Hypertension

(d) HIV status

**13. Indication For Surgery**

(a) Obstructed labour

(b) Previous scar

(c) Foetal distress

(d) APH

(e) Hypertensive Disorders

(f) Others-specify

**14. Preoperative infection**, if yes, which one?___________________________________

**15. Nature of surgery:** (a) Elective (b) Emergency

**16. Timing of surgical antimicrobial prophylaxis**

(1) Before the operation (2) During operation (3) After operation (4) Not initiated at all.

**17. Type of surgical antibiotic prophylaxis given**

(1) Ceftriaxone (2) Metronidazole (3) Both (4) Others

**18. Duration of operation in minutes**

(1) 0-60 (2) 61-120 (3) >120

**19. Family/Social History**

(a)Smoking (b) Alcohol Use

**20. Laboratory Results**

(1) WBC/pus cell seen from G/stain ……………………………………..

(2) Organisms isolated

(a) ……………………………………………..

b) ……………………………………………..

c) ……………………………………………

**22. Sensitivity pattern of isolated organisms**

1…………………………………………….

| Drugs |  |  |  |  |  |  |  |  |  |
| --- | --- | --- | --- | --- | --- | --- | --- | --- | --- |
| Diameter |  |  |  |  |  |  |  |  |  |
| Interpretation |  |  |  |  |  |  |  |  |  |

2………………………….

| Drugs |  |  |  |  |  |  |  |  |  |
| --- | --- | --- | --- | --- | --- | --- | --- | --- | --- |
| Diameter |  |  |  |  |  |  |  |  |  |
| Interpretation |  |  |  |  |  |  |  |  |  |

3…………………………………

| Drugs |  |  |  |  |  |  |  |  |  |
| --- | --- | --- | --- | --- | --- | --- | --- | --- | --- |
| Diameter |  |  |  |  |  |  |  |  |  |
| Interpretation |  |  |  |  |  |  |  |  |  |

4…………………………………..

| Drugs |  |  |  |  |  |  |  |  |  |
| --- | --- | --- | --- | --- | --- | --- | --- | --- | --- |
| Diameter |  |  |  |  |  |  |  |  |  |
| Interpretation |  |  |  |  |  |  |  |  |  |
